# An Accurate Value for the Absorption Coefficient of Silicon at 633 nm

**DOI:** 10.6028/jres.095.043

**Published:** 1990

**Authors:** Jon Geist, A. Russell Schaefer, Jun-Feng Song, Yun Hsia Wang, Edward F. Zalewski

**Affiliations:** National Institute of Standards and Technology, Gaithersburg, MD 20899; Science Applications International Corporation, 4161 Campus Point Court, San Diego, CA 92121; National Institute of Standards and Technology, Gaithersburg, MD 20899

**Keywords:** absorption coefficient, etch stop, extinction coefficient, HeNe, high accuracy, silicon

## Abstract

High-accuracy transmission measurements at an optical wavelength of 633 nm and mechanical measurements of the thickness of a 13-*µ*m thick silicon-crystal film have been used to calculate the absorption and extinction coefficients of silicon at 633 nm. The results are 3105±62 cm^−1^ and 0.01564±0.00031, respectively. These results are about 15% less than current handbook data for the same quantities, but are in good agreement with a recent fit to one set of data described in the literature.

## 1. Introduction

The absorption coefficient [1] *α* at wavelength λ is related to *k*, the imaginary part of the index of refraction, also called the extinction coefficient, by

α=4πk/λ, (1)

where λ is the vacuum wavelength of the incident radiation, which is related to the photon energy *h v* of the radiation by

λhν=1.23985eVμm. (2)

For silicon, *k* spectra have been derived from a Kramers-Kronig analysis of reflectance data [2], by inversion of ellipsometric data [3], and from *α* spectra derived from transmission measurements [4,5]. Neither ellipsometry nor a Kramers-Kronig analysis is well suited to the determination of small values of *k*, so for silicon most *k* values quoted below about 2.5 eV have been determined from transmission measurements. Since the differences among the various data sets are fairly large, Taft [6] measured the absorption coefficient at the Hg 546.1-nm line (2.27 eV) for use in reducing ellipsometric data on oxide films on silicon wafers. However, at this time there is no consensus as to the correct spectra. This is illustrated by the fact that two recent compilations contain spectra that differ by much more than 20% at some wavelengths [7,8].

We have carried out a measurement similar to that of Taft at the 633-nm HeNe laser line. Our purpose was twofold. First, we wanted a smaller uncertainty at 633 nm than could be obtained from any of the published or compiled data. Second, we wanted to test the use of 1) an amplitude-stabilized laser as a radiation source, 2) extremely linear silicon photodiodes as the radiation detector, 3) recently perfected etching techniques for sample preparation, and 4) state-of-the-art mechanical measurements of sample thickness.

The advantage of using amplitude-stabilized lasers in conjunction with extremely linear photo-diodes is that it allows very precise transmission measurements to be carried out over a very large dynamic range with very high wavelength accuracy and resolution. This was demonstrated recently in connection with a search for evidence of the second indirect gap in the silicon absorption coefficient spectrum [9].

The work reported here supplements the high-precision work reported in reference [9] by showing what additional steps are necessary to achieve high accuracy. Combining the techniques described here with those described in reference [9] should allow high-precision, high-accuracy measurements over a large range of transmittance.

There are two reasons why a large range of transmittance is desirable. Since the absorption coefficient of silicon changes by many orders of magnitude over the region of the indirect transition, the dynamic range over which accurate transmittances can be measured determines the number of samples needed to cover the whole spectral range. Second, there are certain advantages that accrue directly from calculating the absorption coefficient from small values of transmittance. The transmission of an optically thick sample is given by the simple expression

$$t=\left(1-r_{f}\right)\;\left(1-r_{b}\right)\;\exp\left(-\alpha \;x\right),$$ (3)

where *r*_f_ and *r*_b_ are the reflectances of the front and back surfaces of the sample, respectively, and *x* is the sample thickness. Therefore,

$$\frac{d\alpha }{\alpha }=-\frac{dx}{x}-\frac{1}{\alpha \;x}\left[\frac{dt}{t}+\frac{dr_{f}}{\left(1-r_{f}\right)}+\frac{dr_{b}}{\left(1-r_{b}\right)}\right].$$ (4)

Thus, the relative uncertainty in *α* will have its minimum value at the largest value of *α x* (smallest transmittance) for which the relative uncertainties in the transmittance and reflectances are independent of the transmittance.

In the following sections, we describe the silicon sample, the optical measurements, the mechanical measurements, the data reduction, and the uncertainty analysis that were used to derive the absorption and extinction coefficient values reported here.

## 2. Silicon Sample

The sample whose transmittance we measured was a 2-cm by 2-cm by 13-*µ*m free standing film of silicon that was prepared as follows. A uniform thickness, p-type, epitaxial layer doped with about 10^14^ B atoms per cm^3^ (50 Ω-cm resistivity) was grown to a nominal thickness of 15 *µ*m on a 100-mm diameter p^+^ (0.01 fl-cm) silicon wafer with (100) surface orientation. A nominal 25- by 25-mm square sample was cut from the wafer, and masked with wax so as to leave the central 2- by 2-cm area of heavily doped substrate exposed. The masked sample was then mounted on a sapphire carrier disk and etched in a rotating-drum mixture of 1 part HF, 3 parts nitric acid, and 8 parts acetic acid [10]. After about 8 h, it was removed and cleaned. The result was a free-standing film, approximately 15-*µ*m thick, suspended in a 0.64-mm thick, 1.9-mm wide silicon frame.

The sample was glued onto a flexible piece of plastic over a 2- by 2-cm hole cut in the plastic so that the etched side faced the plastic. The plastic served as a convenient handle for aligning and mounting the sample, and as a strain relief for the sample during handling. Although the sample was reasonably robust due to the frame, it still could be damaged by rough handling, and the plastic served to minimize stress on it during handling.

## 3. Experimental Measurements

### 3.1 Absolute Transmission and Reflection Measurements

**Figure 1:**
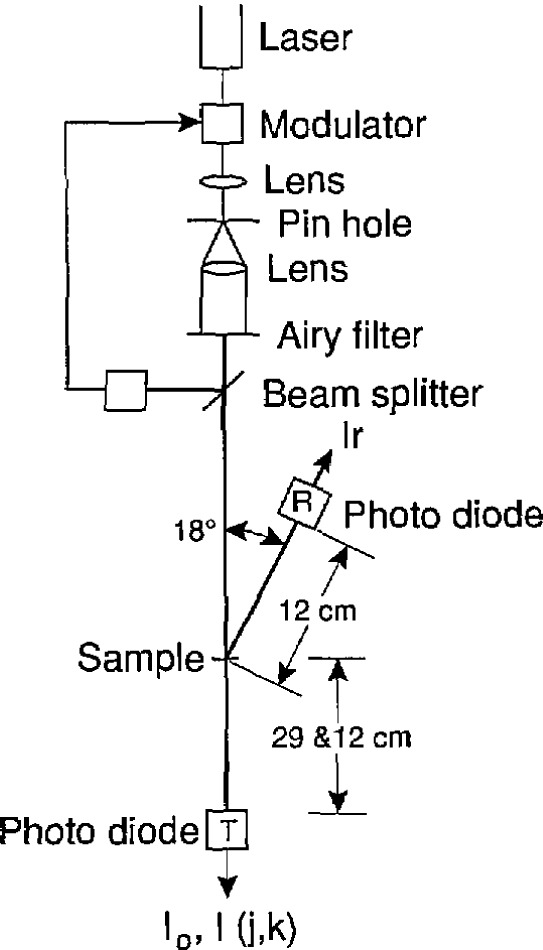
Apparatus for measuring the transmittance and reflectance of the silicon sample at the 633-nm HeNe laser line.

The absolute transmission measurements were carried out using the experimental setup shown in figure 1. A HeNe laser emitting 633-nm radiation was used as the source of radiation. The beam was passed through an electro-optical modulator and focused with a microscope objective onto a pinhole aperture in an opaque screen. The radiation diffracted through the pin hole was collimated with a beam-expanding telescope to produce an Airy disk about 6 mm in diameter. An opaque screen with a 6-mm diameter hole (Airy Filter) was aligned with the first dark ring in the Airy pattern so that only the Airy disk passed through the aperture. This provided a fairly clean and uniform beam of about 40 *µ*W of 633-nm radiation. A beam splitter reflected some of this radiation to a photo-diode whose output controlled the transmittance of the modulator so as to maintain a constant power in the beam to within about 0.1% over a period of hours [11].

The amplitude-stabilized laser beam passed through the sample-holder section of a computer-controlled translation stage and was incident on a photodiode having a 1- by 1-cm active area that was located in position T in figure 1. The photocurrent *I*_0_ from this photodiode was measured with a high-accuracy digital voltmeter and a trans-impedance amplifier. In this and all other photocurrent measurements, the measured value was corrected by subtracting the dark current that was measured with the laser light shuttered and the rest of the apparatus in the same configuration as during the photocurrent measurement.

#### 3.1.1 Direct Transmittance and Reflectance

The plastic sheet to which the sample was attached was positioned on the sample holder such that the laser radiation was incident on the etched side of the sample, and the bottom and top sides of the sample were parallel to one, and perpendicular to another of the directions of motion of the translation stage on which the holder was mounted. The plastic sheet was fastened to the sample holder in this position with removable transparent tape. In this position the photodiode was 29 cm from the sample. The stages were then translated so that the 6-mm diameter HeNe radiation beam was centered on the nominal center of the sample, and the photocurrent *I*_d_(*J, k*), *j* = −1,0, 1, *k* = −1, 0, 1, due to the directly transmitted radiation, was recorded as a function of the position of the sample over a 3 by 3 grid of points on the sample having a 2-mm grid spacing, with the center of the grid pattern coincident with the nominal center of the sample.

The plastic sheet with the sample was removed from the sample holder and remounted as described above, but with the laser radiation incident on the polished surface, and the translation stage rotated about 9° off of the perpendicular to the direction of the laser beam to allow the reflectance of the sample to be measured by the photodiode in position R in figure 1. The resulting photocurrent *I*_r_ reflected from the nominal center of the sample was recorded. The photodiode was relocated to position T in figure 1, but at 12 instead of 29 cm from the sample, and the photocurrent *I* from the radiation directly transmitted through the center of the sample was measured.

The photocurrent *I* was 1.7% greater than *I*_d_(0,0), which was measured at the nominal center of the sample when the etched side of the sample faced the laser and the photodiode was 29 cm from the sample. This difference could be caused by a failure to irradiate exactly the same area of the sample when aligning the laser beam with the nominal center of the sample, or by the different angle of incidence for the different measurements. It could also be caused by slightly more scattered radiation falling on the photodiode when located 12 cm from the sample than when located 29 cm from the sample. In any event, the difference between *I* and *I*_d_(0,0) was not due to laser drift. After the measurement described above, the sample was removed from the sample holder and *I*_0_ was remeasured. The two measurements of *I*_0_ agreed to within 0.1%, indicating the stability of the amplitude-stabilized laser beam.

#### 3.1.2 Scattered Transmittance

**Figure 2:**
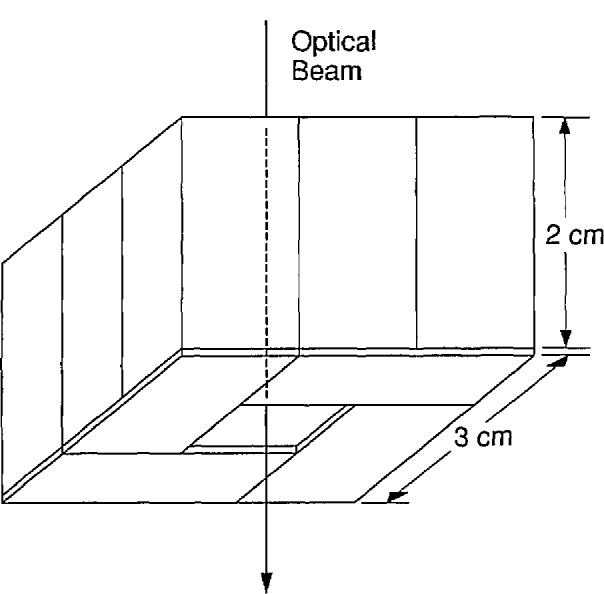
Illustration of the box-shaped scattermeter made from an array of photodiodes to measure the scattered component of the transmitted HeNe laser radiation.

**Figure 3:**
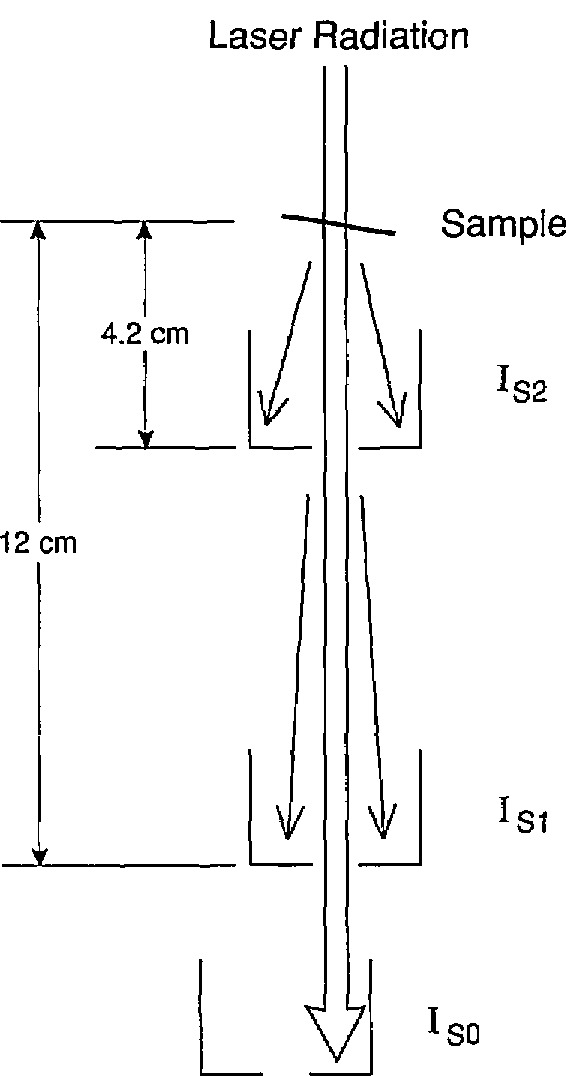
Illustration of the use of the scattermeter shown in figure 2 to measure some scattered components of the transmitted HeNe laser radiation.

A scattermeter package [12] consisting of a box-shaped array of photodiodes with a 1- by 1-cm hole in the center of the array as shown in figure 2 was used to measure the transmitted radiation that was scattered out of the directly transmitted beam. First, the photodiode used to measure the directly transmitted radiation was removed. Then, with the sample still out of the sample holder, the scattermeter was aligned in the beam so that it collected all of the beam on its interior surface, and the photocurrent *I*_s0_ was recorded. The sample was then remounted, as described above, with the etched surface facing the scattermeter. The scattermeter was then located 10 cm from the sample as shown in figure 3, so that the 1- by 1-cm opening in the top of the scattermeter was in the position previously occupied by the photodiode measuring the directly transmitted radiation. The photocurrent *I*_S1_ was recorded. The scattermeter was then moved so that its base was only 2.2 cm from the sample, care being taken to keep the directly transmitted beam in the center of the 1- by 1-cm opening in the top of the scattermeter. The photocurrent *I*_S2_ was recorded. The results, which are *I*_S1_*/I*_0_=0.060% and *I*_S2_/*I*_0_=0.094%, were used in the calculation of the total transmittance as described next.

#### 3.1.3 Transmittance and Reflectance Data Reduction

The transmittance *t*(*j*, *k*), *j*= −1, 0, 1, *k* = −1, 0, 1 of the sample at the same nine points on the 3 by 3 grid where the direct transmittance was measured was calculated as on the assumption that the transmittance of the sample was independent of whether the radiation was transmitted from the etched surface to the polished surface, or vice versa, and that the transmitted radiation that was scattered scaled with the directly transmitted radiation. The *t*(*j*, *k*) values calculated from eq (5) are listed in table 1.

$$t\left(j,k\right)=\frac{I_{d}\left(j,k\right)}{I_{d}\left(0,0\right)}\left[\frac{I}{I_{0}}+\frac{I_{S1}+I_{S2}}{I_{S0}}\right],$$ (5)

The polished side of the sample, which faced the laser beam, was smooth enough to reflect specularly with negligible scattering. Moreover, when the radiation was incident on the polished side of the sample, the fraction that was reflected from the etched side was attenuated by (1 *− r*)^2^ exp(−2 *α x*)<2 × 10^−4^ as it was transmitted through the polished face of the sample to the etched face, and back again. Therefore, the scattered component of radiation reflected from the etched surface was negligible compared to the radiation reflected specularly from the polished surface, and no attempt was made to measure it.

The reflectances of both faces of the sample were calculated as

$$r_{b}=r_{f}=I_{r}/I_{0}=0.3454,$$ (6)

under the assumption that the roughness of the etched surface was not large enough to change the reflectance of the surface significantly, even though it was large enough to scatter a significant amount of radiation out of the directly transmitted beam. How this assumption was tested is described in the next section.

### 3.2 Mechanical Measurements

#### 3.2.1 Surface Roughness

**Figure 4:**
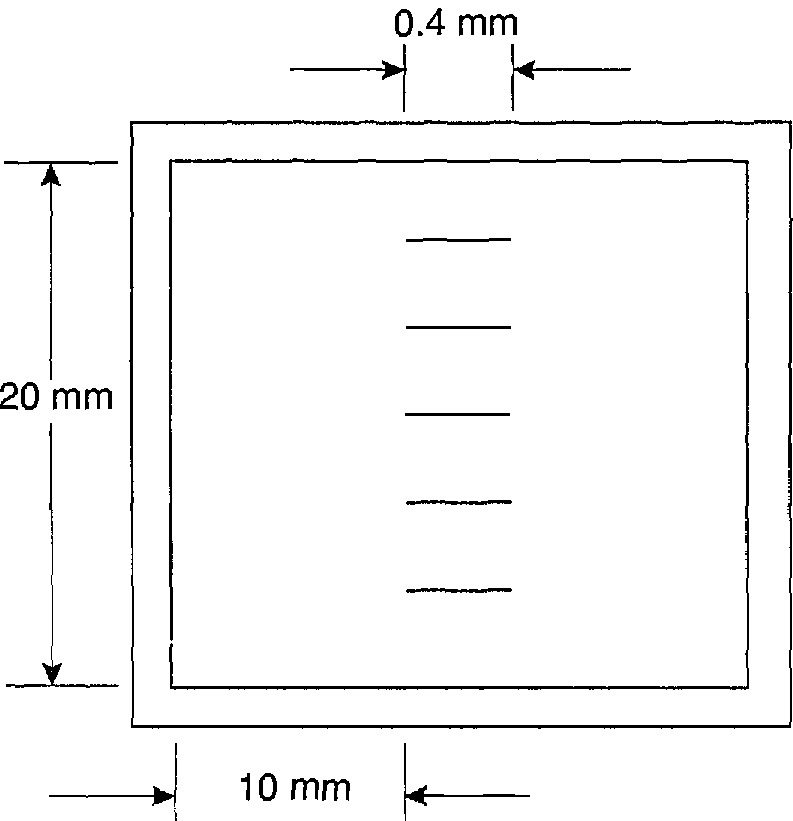
Illustration of the location on the sample of the surface roughness measurements. Note that the length of the surface roughness scans is exaggerated in this figure.

The characterization of the roughness of the etched surface of the sample was carried out by measuring the roughness along five lines located on the sample as shown in figure 4 using the NIST Stylus/Computerized System [13], in which a high-resolution TALYSTEP^2^ stylus instrument is calibrated on the 200,000 × vertical magnification range against a 0.0291-*µ*m step-height standard that was measured interfero-metrically. To allow the fine structure of the surface to be resolved, a stylus width of 0.15 *µ*m with a loading of 0.15 mgf (1.5 *µ*N) was used. The TALYSTEP ISO filter (2CR) was used, and the cut-off wavelength was 0.0976 mm (25% attenuation). The traversing speed was 0.122 mm/s over a distance of 0.4 mm. Since the instrument’s high-frequency response is 85 Hz, the surface profile could be detected over a wavelength range extending from about 1.44 *µ*m (corresponding to the frequency response of the instrument) to 97.6 *µ*m (the filter cut-off wavelength). The root-mean-square surface roughnesses *R*_q_ over this wavelength range varied from 7.2 to 10.0 nm for the five lines, while the arithmetic average roughness *R*_a_ varied from 6.3 to 8.1 nm. This is not large enough to disturb the mechanical thickness measurements described next, nor to change the reflectance of the surface, but it is large enough to scatter both transmitted and reflected radiation significantly out of the respective direct and specular directions.

#### 3.2.2 Sample Thickness

We used a high-accuracy laser-interferometer micrometer (GCA LASERULER) having a resolution of 0.01 *µ*m and an uncertainty of ±0.13 *µ*m to measure the thickness of the sample. For these measurements, the micrometer was fitted with a 0.2-in (5.1-mm) diameter, hemispherical, steel-tipped probe, and a steel surface plate of nominal 130-mm diameter was centered under the probe. The digital output from the laser-interferometer that was connected to the probe shaft was set to zero when the probe was driven against the surface plate with the probe-driving mechanism. The probe was then raised from the surface plate and driven against it, and the zero-point height recorded six times. The probe was again raised, the sample placed on the surface plate with its polished surface down, and the probe driven to within a millimeter of the etched surface. In this position, the sample location was adjusted to be nominally centered under the probe as determined by sighting along the diagonals of the sample.

The probe was then lowered and raised six times, and the sample thickness was recorded each time. The zero-point thickness measurements were then repeated six times. The thickness *h* was calculated from the difference of the mean of the thickness measurements and the mean of zero-point measurements. The value determined in this way was *h* = 12.86 ± 0.02 *µ*m, where the stated uncertainty is one standard deviation of the reported value. The purpose of this value was to serve as a consistency check on the data on the variation of thickness over the sample that is described next.

A small piece of graph paper was fastened to the surface plate with removable transparent tape in such a way that one corner of the plastic sheet to which the sample was fastened fell in the center of the graph paper when the sample was nominally centered under the micrometer probe. The graph paper did not extend under the sample for any position of the sample that allowed the probe to touch the etched surface of the sample. The plastic sheet was then aligned so that one edge was parallel to one of the lines on the graph paper, one corner was aligned with a point where horizontal and vertical lines on the graph paper crossed, and the micrometer probe was located over the etched portion of the sample near one corner. The plastic sheet was then translated over an 8 by 8 grid with a 2-mm grid spacing and the height *z*(*m,n*), *m* = 0, …, 7, *n* =0, …, 7, of the sample measured at each grid point. After the measurement of *z*(*m,n*) at each grid point, the plastic sheet was returned to the starting point (0,0) and the thickness *z*(0,0;*m,n*) was remeasured to monitor indirectly any zero drift in the digital readout of the micrometer.

When the measurements described above were completed, the plastic sheet was removed and a zero-point reading was taken. The zero-point reading had drifted by 0.32 *µ*m during the set of 64 grid point and 64 reference-point measurements, but the reference-point measurements indicated that it had reached even larger values a few times during the measurements. Therefore, the reference-point measurements were used in making the zero-point corrections to the thickness map data.

The plastic sheet was then alternately aligned at the point (1,3) and removed three times, so that three measurements of sample thickness followed by three measurements of the zero-point value could be recorded. The thickness *h*(1,3) = 12.79±0.00 *µ*m of the sample at the point (1,3) was calculated as the mean of the sample thickness measurements minus the mean of the zero-point readings. The uncertainty was calculated in the same way as it was for the measurement at the nominal center of the sample that was described at the beginning of this section.

Finally, the thickness *h*(*m,n*) of the sample at each grid point was calculated as

$$\begin{array}{c} h\left(m,n\right)=h\left(1,3\right)+\left[z\left(m,n\right)-z\left(0,0;m,n\right)\right]\\ \;-\left[z\left(1,3\right)-z\left(0,0;1,3\right)\right]. \end{array}$$ (7)

**Figure 5:**
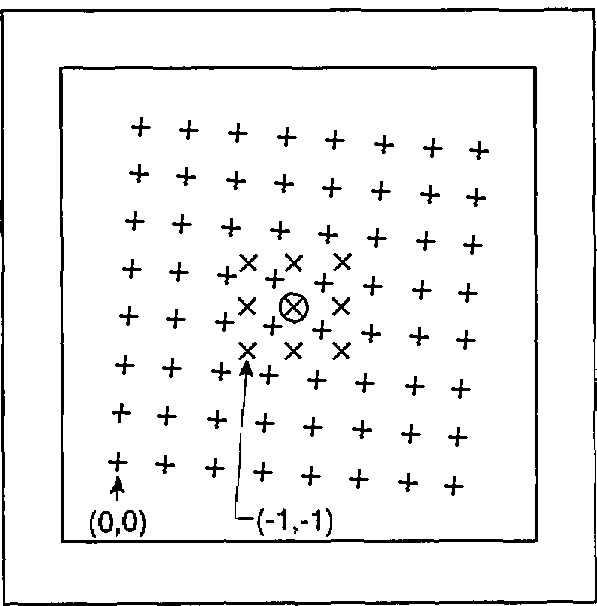
Approximate relative locations of the thickness measurements (+) and the transmittance measurements (×) relative to the center (circled ×) of the sample.

Table 2 shows the *h*(*m,n*) data obtained from eq (7). Figure 5 shows as crosses the approximate location of the 8 by 8 grid where the *h*(*m,n*) data were measured on the sample as determined by observing the location of the sides of the micrometer probe relative to the corners of the sample after the measurement described above was completed. For comparison, figure 5 also shows as ×’s the nominal location of the transmittance measurements reported earlier in this paper. The circled × is the point (0,0) for the transmittance data, and it is also the nominal location of the thickness measurement reported earlier in this section as a consistency check. That value, *h* = 12.86±0.02 *µ*m, agrees to within 0.1 *µ*m with the expected value on the basis of the data shown in table 2.

#### 3.2.3 Elastic Compression Correction

**Figure 6:**
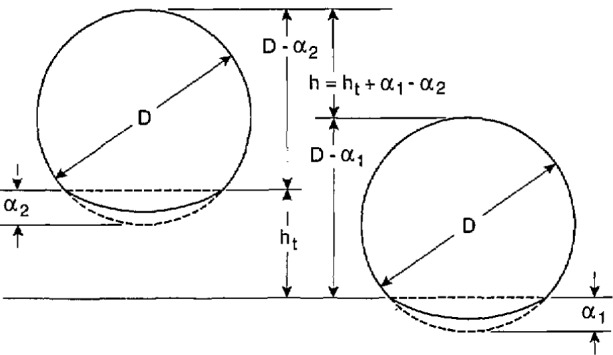
Illustration showing the relation between the true thickness *h*_t_, of the sample and the measured thickness *h* due to the difference in compression between the sample-in and sample-out measurements with the laser micrometer.

All of the silicon thicknesses reported so far contain an error due to the elastic compression of the hemispherical probe tip and the surface that it contacts under the load of a thickness or zero-reference measurement. The true thickness of the sample *h*_t_ is related to the difference *h* between the sample-in and sample-out measurement by *h*_t_ = *h* +*Δh*, where *Δh* = *α*_2_*−α*_1_, as is illustrated in figure 6. To calculate *Δh*, we used the equation for the elastic compression *α*_i_, of a sphere in contact with a flat surface,

$$\alpha_{i}=\frac{{\left(3\;\pi \;p\;[V_{\mathrm{flat}}+V_{\mathrm{sphere}}]\right)}^{2/3}}{2\;D^{1/3}},$$ (8)

where *p* is the total applied force, *D* is the diameter of the sphere, and

$$V_{j}=\left(1-\sigma_{j}^{2}\right)/\left(\pi \;E_{j}\right),$$ (9)

where σ*_j_* is Poisson’s ratio of the *j*th material, and *E_j_* is the modulus of elasticity of they *j*th material.

Reference [14] gives 0.956×10^−8^ in^2^/lbf (1.39 pm^2^/N) for *V*_steel_ References [15] and [16] give 0.44 and 1.56×10^6^ lbf/in^2^ (3.0 and 10.8 GN/m2) for σ_Si_ and *E*_Si_, respectively, without indicating the crystallographic direction to which the values actually apply. For forces applied to a (100) surface silicon, *E*_Si_ is given by

$$E_{\mathrm{si}}=\left(C_{11}+2\;C_{12}\right)\cdot \left(C_{11}-C_{12}\right)/\left(C_{11}+C_{12}\right),$$ (10)

where *C_kl_* are the tensor stiffness constants of silicon [17]. Reference [18] gives values for *C_kl_* from which we calculated *E*_Si_=18.9×10^6^ lbf/in^2^ (130 GN/m^2^). The diameter of the tip of the hemispherical probe was mea11111111sured to be 0.20 in (5.1 mm), and the force applied during a thickness measurement was measured to be 0.154 lbf (0.658 N). Using the above values, we calculated *Δh* =0.035 *µ*m and assigned plus or minus the calculated value as a conservative uncertainty.

## 4. Data Reduction and Uncertainty Analysis

### 4.1 Absorption Coefficient

**Figure 7:**
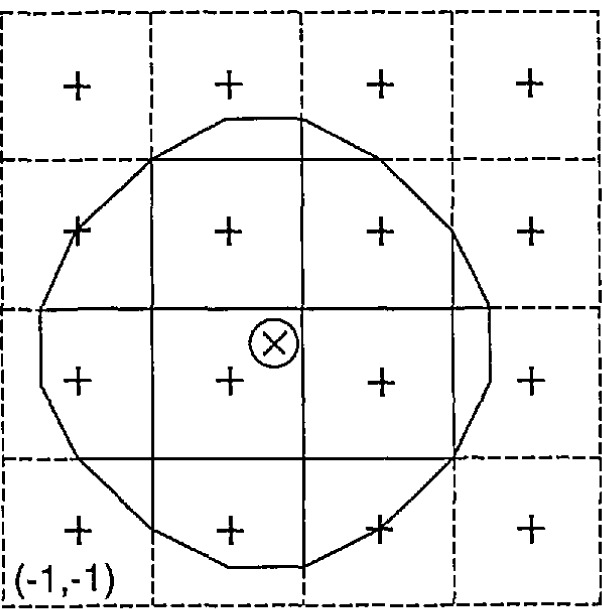
Illustration of the averaging of the thickness measurements to approximate the same weighting over the 6-mm diameter region as obtained from the transmittance measurements. This weighting is appropriate for the center of the sample, but is a little skewed relative to other locations of the transmittance measurements on the sample.

An absorption coefficient value was calculated for each of the transmittance measurements by inverting eq (3) with *r*_f_ *= r*_b_=0.3454, as indicated in eq (6), to obtain

$$\alpha \left(j,k\right)=\frac{-\ln\left\{t\left(j,k\right)/\left[\left(1-r_{f}\right)\left(1-r_{b}\right)\right]\right\}}{x\left(j,k\right)+\Delta h},$$ (11)

where

$$\begin{array}{l} x\left(j,k\right)=\\ \frac{\left[\sum_{m=j+2}^{m=j+5}\sum_{n=k+2}^{n=k+5}w\left(m-j-3,n-k-3\right)h\left(m,n\right)\right]}{108}, \end{array}$$ (12)

for *j* = −1, 0, 1, *k* = −1, 0, 1, and where the weights *w*(*u, v*) for *u* = −1, 0, 1, 2, *v*= −1, 0, 1, 2 are listed in table 3. These weights were chosen as illustrated in figure 7 to average the measured sample thickness over approximately the same 6-mm diameter area in the center of the sample as was used in the transmittance measurements. Table 4 lists the resulting average thicknesses *x*(*j*, *k*), and table 5 lists the absorption coefficient values obtained from eq (11).

### 4.2 Uncertainty Analysis

The uncertainty in the absorption coefficient can be calculated from the total differential in eq (4). First, we consider the sources of error that are common to every point (*J*, *k*). These sources of error include systematic radiometric errors in the direct and scattered transmittance measurements, and in the reflectance measurement, uncertainties associated with using the reflectance measured from the polished side of the sample to represent that from the etched side, and with using the sum of the measured scattered transmittances to represent the true scattered transmittance. Inaccuracies in the laser micrometer and the calculation of the depth *Δh* to which the micrometer probe penetrates into the silicon during the thickness measurement are also sources of error common to all of those measurements. These sources of error and our uncertainty estimates for them are listed in table 6. Also listed there is the quadrature sum of their contributions to the relative uncertainty in the absorption coefficient.

Referring to table 6 and eq (4), recall that the measured sample thickness was approximately 13 *µ*m. Since the uncertainty associated with the laser micrometer is 0.13 *µ*m, it contributes 1*%* to the uncertainty in the absorption coefficient. Similarly, the 0.035-*µ*m uncertainty assigned to the compression of the silicon, the surface plate, and the probe tip contributes 0.27%.

There is a 1% uncertainty associated with the transmittance-photocurrent ratios because the gain of the current-to-voltage converter was 100 times greater for the measurements of the numerator photocurrents than for the denominator photocurrents, and 1% is the between-range gain accuracy of the transimpedance amplifier used. An uncertainty of 25% of the measured components of the scattered transmittance was associated with the use of the sum of these quantities for the true scattered transmittance. Since the sum of the measured components of the scattered transmittance was about 15% of the total measured transmittance, the relative uncertainty is about 3.8%. An uncertainty of less than 0.1% is associated with the reflectance ratio since the same gain was used for both the numerator and the denominator photocurrents in this measurement. However, an uncertainty of 1% was associated with *r*_b_ to allow for the possibility that it was smaller than *r*_f_ due to the roughness of the etched surface. According to eq (4), each of these uncertainties is multiplied by a factor of 1/*αx* (about 1/4 for this sample and wavelength) in its contribution to the uncertainty in the absorption coefficient, as shown in table 6.

Sources of error that contribute differently to the absorption coefficient values at the different points (*j*, *k*) are the random errors in the measurements of the transmittance, reflectance, and thickness. The first two are negligible at the 0.1 % level. The third might not be, but its actual contribution to our data cannot be distinguished from the error associated with the variation in thickness of the sample as a function of position over its surface.

It is not really clear how to estimate the uncertainty to be associated with the variations in sample thickness. On the one hand, the thickness averaging procedure was designed to eliminate errors from this source. On the other hand, the procedure is not perfect because the transmittance values and the average thicknesses do not weight the same parts of the sample in the same way. The transmittance values weight the points of the sample within a 6-mm diameter region according to the intensity of the Airy disk. The average thicknesses weight 13 points on a 2- by 2-mm grid nonuniformly to approximate equal weighting of all points in a 6-mm diameter region. Furthermore, the centers of the corresponding regions over which the transmittance and thickness averages were computed are only approximately aligned.

### 4.3 Reported Value and Uncertainty

After trying a number of different approaches to the uncertainty analysis, we decided to use the average of the *α*(*J*, *k*) values in table 5 for the reported value, and to use the half-range of the values as the uncertainty. This results in a contribution of 0.76% to the relative uncertainty in the absorption coefficient. When we add this relative uncertainty in quadrature with the relative uncertainties reported in table 6, we obtain 1.94%. Therefore, we report *α* = 3105 cm^−1^ and *k* =0.01564 with an estimated uncertainty of 2% for silicon at a vacuum wavelength of 633.00 nm or a photon energy of 1.9587 eV.

### 4.4 Comparison with Existing Data

**Figure 8:**
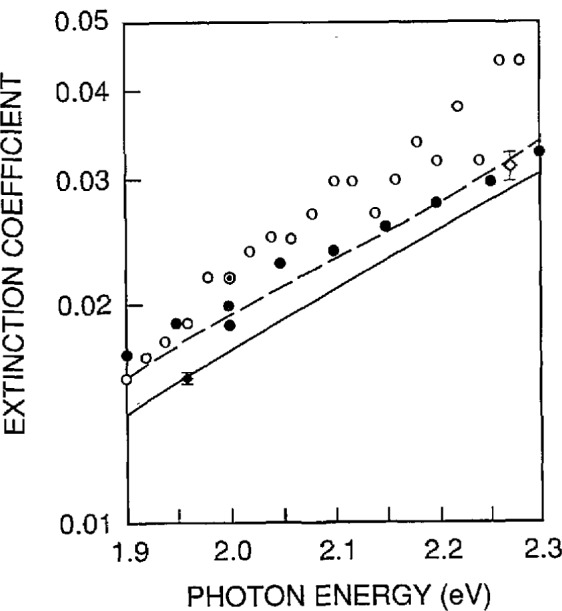
Comparison of Handbook data (open circles from reference [7] and closed circles from reference [8]), published data reported without tabulation (dashed line from reference [4] and continuous line from reference [5]), and single-point transmittance measurement (open diamond from reference [6]) with the value reported at 633 nm in this paper.

Figure 8 compares our data at 1.96 eV with some of the more noteworthy data previously reported in the 1.9- to 2.3-eV spectral region. The open circles are the handbook data of reference [7] and the closed circles are the handbook data of reference [8]. The dashed line is the data (for which no tabulated values were published) of reference [4], and the full line is data (for which no tabulated values were published) of reference [5]. The open diamond is the value reported in reference [6], along with the reported uncertainty. Our value is the closed diamond.

The result reported here has an estimated uncertainty that is significantly smaller than the other results shown in figure 8, due to combining a higher quality sample with more accurate and precise thickness and transmittance measurements. Two sources of uncertainty dominate the uncertainties listed in table 6. These are the measurement of the thickness of the sample and the measurement of the scattered component of the transmittance. It should be possible to improve both of these measurements to reduce the overall uncertainty to below 1%, either by using more accurate instrumentation, and/or by improving the sample quality. In either case it would be necessary to obtain a more accurate laser micometer. Other required improvements in instrumentation would include a more accurate scattermeter, and a precision translator that clamps onto both the surface plate used in the thickness measurements and the sample holder used in the transmittance measurements. The latter would allow precise alignment of the areas weighted by the transmittance and thickness measurements, as well as a finer density of points for the thickness measurements. Improvements in the sample preparation procedure that resulted in a sample having a more uniform thickness and a smoother rear surface would eliminate the requirement for the improved scattermeter and the translation stage. However, at the current time, it is not clear how to obtain the necessary improvements in sample preparation.

## Figures and Tables

**Table 1:** The absolute transmittance *t*(*j*, *k*) measured on the silicon sample described in this paper

*t*(*j*,*k*)			
${\;}_{k}\backslash^{j}$	−l	0	1
1	0.00802	0.00778	0.00824
0	0.00786	0.00783	0.00816
−1	0.00766	0.00768	0.00800

**Table 2:** The thickness *h* (*m*, *n*) of the sample at points located at the approximate positions indicated in figure 5

*h*(*m,n*) in *µ*m								
${\;}_{n}\backslash^{m}$	0	1	2	3	4	5	6	7
7	10.97	11.59	11.49	11.99	12.11	11.75	13.29	10.70
6	12.42	12.66	12.80	12.58	12.60	12.56	12.24	11.68
5	12.89	12.82	13.15	12.83	12.44	12.66	12.76	11.93
4	12.94	13.01	12.79	12.81	12.54	12.80	12.88	11.84
3	12.97	12.79	12.71	12.96	12.81	12.79	12.85	11.80
2	12.95	13.05	12.95	12.66	13.02	12.88	12.50	11.68
1	12.95	12.90	13.05	13.14	12.73	12.58	12.56	11.59
0	12.17	12.61	12.68	12.74	12.55	12.30	11.97	11.36

**Table 3:** The weights *w*(*u,v*) used in calculating average thickness of the sample over the regions irradiated during the transmittance measurements

108·*w*(*u*,*v*)				
${\;}_{u}\backslash^{v}$	−1	−0	1	2
2	0	3	1	0
1	7	16	14	1
0	11	16	16	3
−1	2	11	7	0

**Table 4:** The average thickness *x*(*J, k*) of the sample over the same areas for which the transmittance data are shown in table 1

*x*(*j*,*k*) in *µ*m			
${\;}_{k}\backslash^{j}$	−1	0	1
−1	12.87	12.75	12.69
0	12.84	12.78	12.79
1	12.89	12.88	12.81

**Table 5:** The absorption coefficients *a*(*j*,*k*) of silicon at 633 nm determined from the data in tables 1 and 4

*a*(*j*,*k*) in cm−1			
${\;}_{k}\backslash^{j}$	−1	0	1
1	3114	3106	3090
0	3114	3123	3128
−1	3099	3089	3081

**Table 6:** The sources of error and associated uncertainty estimates for the sources of error that are common to the thickness and transmittance measurements at all points on the sample

Source of error	Estimated uncertainty	
*dx/x*		times 1
Micrometer accuracy	1.00%	1.00%
Compression correction	0.27%	0.27%
*dt/t*		times 1/*αx*
*I*_d_(*j*,*k*)/*I*_d_(0,0)	1.0%	0.25%
*I/I*_0_	1.0%	0.25%
*I*_S1_*/I*_S0_	1.0%	0.25%
*I*_S2_*/I*_S0_	1.0%	0.25%
*(I*_S1_*/I*_S2_)/*I*_s0_ for true scatter	3.8%	0.95%
d*r*_f_/(1−*r*_f_)		times 1/*αx*
*I*_r_*/I*_0_	<0.1%	0.02%
d*r*_b_/(1 − *r*_b_)		times 1/*αx*
Use of *r*_f_ for *r*_b_	1.0%	0.25%

Sum in quadrature		1.79%

## Footnotes

2 Certain commercial equipment, instruments, or materials are identified in this paper to specify adequately the experimental procedure. Such identification does not imply recommendation or endorsement by the National Institute of Standards and Technology, nor does it imply that the materials or equipment identified are necessarily the best available for the purpose.
